# Co-infection VIH chez les tuberculeux suivis au service des maladies infectieuses du CHU Ibn Rochd-Casablanca

**DOI:** 10.11604/pamj.2018.30.276.13913

**Published:** 2018-08-16

**Authors:** Assiya El Kettani, Sanae Jebbar, Brahim Takourt, Fakhreddine Maaloum, Othman Diraa, Brahim Farouqi, Khalid Zerouali, Kamal Marhoum El Filali

**Affiliations:** 1 Laboratoire d’Immuno-Sérologie, CHU Ibn Rochd-Casablanca, Maroc; 2 Service des Maladies Infectieuses, CHU Ibn Rochd-Casablanca, Maroc; 3 Laboratoire de Bactériologie-Virologie et Hygiène, CHU Ibn Rochd-Casablanca, Maroc

**Keywords:** Co-infection, tuberculose, infection par le VIH, prévention, Co-infection, tuberculosis, HIV co-infection, prevention

## Abstract

L'objectif du travail est de déterminer la prévalence de l'infection par le VIH chez les tuberculeux hospitalisés au service des maladies infectieuses du CHU Ibn Rochd-Casablanca et de chercher les facteurs associés à la co-infection Tuberculose-VIH. Nous avons mené une étude transversale rétrospective menée en Novembre 2016, en se basant sur l'exploitation de la base de données du service des maladies infectieuses, des laboratoires de microbiologie et d'immunologie du CHU Ibn Rochd. Etaient inclus les patients ayant une tuberculose confirmée par culture positive sur milieu de Lowenstein Jensen et une infection à VIH confirmée par Western Blot entre Janvier 2013 et Décembre 2015. Au cours de la période de l'étude, 117 cas de tuberculose ont été confirmés par culture. Parmi ces patients, 46 (39,3%) avaient une infection à VIH confirmée. Trente-quatre patients co-infectés (73,9%) avaient une tuberculose extra pulmonaire (cette forme était associée à la co-infection (p=0,04)). Tous les patients ont été mis sous traitement antituberculeux et antirétroviral selon l'indication. L'évolution était favorable chez 32 patients (69,6%) et 10 décès ont été enregistrés (21,7%). La mortalité était plus élevée chez les co-infectés que chez les tuberculeux non atteints par le VIH (8,4%), (p=0,04). Cette étude a objectivé une prévalence relativement élevée de l'infection par le VIH parmi les tuberculeux. La co-infection Tuberculose-VIH est associée aux formes graves de la tuberculose et à une augmentation de la mortalité chez les tuberculeux, d'où l'intérêt du renforcement des activités conjointes de lutte contre cette co-infection.

## Abstract (en)

This study aims to determine the prevalence of HIV infection among patients hospitalized for tuberculosis in the Division of Infectious Diseases at the Ibn Rochd University Hospital in Casablanca and factors associated with TB-HIV co-infection. We conducted a cross-sectional retrospective study in November 2016. The database of the Division of Infectious Diseases, Laboratory of Microbiology and Immunology at the Ibn Rochd University Hospital was examined. All the patients with tuberculosis confirmed using Lowenstein Jensen culture medium and HIV Infection confirmed using Western Blot test between January 2013 and December 2015 were included. During the study period, 117 cases of tuberculosis were confirmed by culture. Fourty six (39.3%) patients had confirmed HIV infection. Thirty-four co-infected patients (73.9%) had extrapulmonary tuberculosis (this form was associated with co-infection (p=0.04)). All patients underwent TB treatment and anti-retroviral treatment according to the indication. The Evolution was favorable in 32 patients (69.6%) and 10 deaths were recorded (21.7%). Mortality rate was higher in co-infected patients than in TB patients without HIV (8.4%), (p=0.04). This study highlights a relatively high prevalence of HIV infection among tuberculosis patients. TB-HIV co-infection is associated with severe forms of tuberculosis and with an increase in mortality rate among TB patients; hence the importance of strengthening anti-co-infection joint activities.

## Aux editeurs du Journal Panafricain de Médecine

La tuberculose (TB) infecte une personne sur trois dans le monde, soit plus de 2 milliards de sujets infectés [1]. Quoique la majorité des personnes infectées ne développent pas la maladie, l'OMS estime qu'en 2015 il y a eu 10,4 millions de cas de tuberculose maladie et 1,79 million de personnes sont décédées de tuberculose [2, 3]. Tous les pays sont affectés, mais 85% des cas sont recensés en Afrique (30%) et en Asie (55%). Dans les années 1980, avec l'avènement du VIH, il y a eu émergence de la tuberculose comme maladie opportuniste associée au sida [4]. La tuberculose quant à elle augmente la réplication virale chez les personnes infectées par le VIH et accélère la progression de la maladie. En 2015, 11% des nouveaux cas de tuberculose (approximativement 1.2 million) dans le monde, avaient une coinfection HIV/TB et 390.000 décès ont été recensés [2, 3] Selon le programme Halte à la tuberculose et VIH/sida de l'OMS, la surveillance des co-infections TB-VIH est recommandée pour planifier des activités conjointes de lutte contre la Tuberculose et l'infection par le VIH [5]. L'objectif du travail est de déterminer la prévalence de l'infection par le VIH parmi un échantillon de patients tuberculeux, diagnostiqués dans le service des maladies infectieuses du CHU Ibn Rochd de Casablanca et de chercher les facteurs associés à la co-infection TB-VIH. Nous avons mené une étude transversale en novembre 2016 concernant les patients, hospitalisés au service des maladies infectieuses du CHU Ibn Rochd, ayant une tuberculose confirmée par culture positive sur milieu de Lowenstein Jensen et une infection à VIH confirmée par Western Blot entre janvier 2013 et décembre 2015.

La collecte de données était basée sur l'exploitation de la base de données du service des maladies infectieuses, des laboratoires de microbiologie et d'immunologie du CHU. Les facteurs associés à la co-infection ont été recherchés. Le test khi 2 a été utilisé pour comparer les pourcentages et le seuil de signification a été fixé à 0,05. L'analyse statistique était réalisée sur Epi info 7. Au cours de la période de l'étude s'étalant de janvier 2013 à décembre 2015, 117 cas de tuberculose ont été confirmés par culture sur milieu de Lowenstein Jensen. Tous ces patients ont eu un dépistage du VIH au moment du diagnostic et 46 (39,3%) avaient une infection à VIH confirmée par Western Blot. En ce qui concerne les patients co-infectés, la tranche d'âge prédominante était celle de 30ans à 39ans (52,2%), avec une prédominance masculine à 63%. La tuberculose extrapulmonaire était notée chez 74,5% des patients co-infectés et était significativement associée à la co-infection (p=0,04). La bascilloscopie était négative chez 73,5% des patients co-infectés. Tous les patients ont été mis sous traitement antituberculeux et seulement 93,5% sous traitement antirétroviral du fait de la mortalité précoce. L'évolution était favorable avec une augmentation du nombre des CD4 pour 32 patients (69,6%). Dix décès ont été enregistrés (21,7%) et 4 patients ont été perdus de vue. La mortalité chez les patients co-infectés était plus élevée que chez les tuberculeux indemnes de l'infection par le VIH (21,7% vs 8,4%) (p=0,04). Tableau 1Cette étude a objectivé une prévalence relativement élevée de l'infection par le VIH parmi les tuberculeux (39,3%); Ce qui est légèrement plus élevé que les résultats de certaines études antérieures (27,7% au Brésil, 26,06% au Cameroun) [6, 7]. Ceci pourrait être expliqué par le fait que notre travail s'est limité à un service des maladies infectieuses, chose qui pourrait surestimer le nombre de co-infections retrouvées.

**Tableau 1:** Les facteurs associés à la co-infection TBK-VIH

Facteurs	TBK-VIH		TBK		p-value
	**N**	**%**	**N**	**%**	
**Sexe**					0,9
Homme	30	65,2	46	64,8	
Femme	16	34,8	25	35,2	
**Age**					0,04
18ans-29ans	3	6,5	12	16,9	
30ans-39ans	24	52,2	38	53,5	
40ans-49ans	13	28,3	19	26,8	
>50ans	6	13	2	2,8	
**Forme clinique de la tuberculose**					0,04
Pulmonaire	14	30,4	35	49,3	
Extra pulmonaire	32	69,6	36	50,7	
**Bascilloscopie**					0,6
Positive	12	26,1	16	26,2	
Négative	34	73,9	55	73,8	
**Evolution sous traitement**					0,04
Favorable	32	65,3	60	84,5	
Défavorable	10	21,7	11	15,5	

La co-infection VIH-Tuberculose était plus fréquente chez les jeunes de 30 à 39 ans. Elle était associée aux formes graves de la tuberculose (Tuberculose extrapulmonaire) et à une augmentation du taux de mortalité chez les tuberculeux; ce qui rejoint les résultats des études antérieures [6, 7]. Par ailleurs, les indicateurs de la co-infection recherchés dans cette étude rejoignent les cibles du plan mondial Halte à la tuberculose 2011-2015 [8], sauf pour ce qui est du pourcentage des patients ayant reçu un traitement antirétroviral qui était de 93,9% au lieu de 100%. Les patients n'ayant pas reçu de traitement antirétroviral sont décédés dans les deux semaines après le début du traitement antituberculeux. En effet, selon les lignes directrices publiées en 2015 par l'OMS pour la prévention du VIH/sida, le traitement antirétroviral est recommandé pour toutes les personnes atteintes du VIH/sida dès que possible mais après le diagnostic de tuberculose, les individus devraient initier le traitement antirétroviral dans les deux à huit semaines pour éviter les interactions entre les traitements antirétroviraux et les traitements antituberculeux et le risque d'hyper activation immunitaire [9]. Le fait que la bacilloscopie ait été positive chez seulement 26,1% des co-infectés et 26,2% des tuberculeux (toute forme confondue) était une limite de l'examen direct. L'examen d'expectorations, après la coloration de Ziehl-Neelsen, bien que considéré comme une technique de référence parmi les examens directs, est moins sensible que la culture d'une part et d'autre part, la positivité de la bacilloscopie est moins fréquente chez les sujets infectés par le VIH avec un taux de CD4 inférieur à 200/mm^3^ [10]. Il est à noter qu'en fin 2015 le laboratoire de microbiologie du CHU Ibn Rochd de Casablanca a mis en place des techniques moléculaires de diagnostic rapide de mycobactéries (notamment la PCR en temps réel). De plus, il s'est renforcé d'un système de management de la qualité; ce qui rejoint les recommandations du plan mondial Halte à la tuberculose 2011-2015 [8]. Néanmoins, Les mesures de prévention de la co-infection TBK-VIH devraient s'intensifier encore plus en améliorant l'accès au continuum des soins via des plates formes de suivi commun ainsi que l'intégration des déterminants socio-économiques dans la lutte contre la tuberculose et l'infection par le VIH et la recherche des facteurs de prédisposition génétique à la tuberculose et aux infections associées.

## Conclusion

La prévalence de l'infection par le VIH chez les tuberculeux reste encore élevée dans notre contexte, en dépit des mesures prises pour lutter contre cette co-infection. Outre le renforcement des mesures de préventions relatives aux prestations de dépistage, de diagnostic, de traitement et de suivi, la lutte devrait également considérer les facteurs socio-économiques ainsi que les déterminants génétiques associés à cette co-infection.

## Conflits d’intérêts

Les auteurs ne déclarent aucun conflit d'intérêts.

## References

[1] World Health Organization Tuberculosis.

[2] WHO Global Tuberculosis Report 2016.

[3] World Health Organization HIV-associated tuberculosis.

[4] World Health Organization (2012). WHO policy on collaborativeTB/HIV activities: guidelines for national programmers and other stakeholders.

[5] Guide de suivi et d'évaluation des activités conjointes tuberculose/VIH, Révision 2015 OMS.

[6] Castro Castrighini C, Reis RK, Souza Neves LA, Galvão MTG, Gir E (2013). Epidemiological profile of HIV/Tuberculosis co-infection in a City in the State of São Paulo, Brazil. J Antivir Antiretrovir.

[7] Noubom M, Nembot FD, Donfack H, Mfin PSK, Tchasse F (2013). Caracterisitiques des patients tuberculeux à l'ouest cameroun: 2000-2009. Pan Afr Med J.

[8] El Kamel A, Joobeur S, Skhiri N, Cheikh Mhamed S, Mribah H, Rouatbi N (2015). La lutte antituberculeuse dans le monde. Rev Pneumol Clin. Avril.

[9] World Health Organization (2015). Guideline on when to start antiretroviral therapy and on pre-exposure prophylaxis for HIV.

[10] Padmapriyadarsini C, Narendran G, Swaminathan S (2011). Diagnosis & treatment of tuberculosis in HIV co-infected patients. Indian J Med Res.

